# First‐line immunotherapy or angiogenesis inhibitor combined with chemotherapy for advanced non‐small cell lung cancer with *EGFR* exon 20 insertions: Real‐world evidence from China

**DOI:** 10.1002/cam4.4852

**Published:** 2022-05-24

**Authors:** Guangjian Yang, Yaning Yang, Runze Liu, Weihua Li, Haiyan Xu, Xuezhi Hao, Junling Li, Shuyang Zhang, Fei Xu, Siyu Lei, Yan Wang

**Affiliations:** ^1^ Department of Respiratory Medicine Shandong Cancer Hospital and Institute, Shandong First Medical University and Shandong Academy of Medical Sciences Jinan Shandong China; ^2^ Department of Medical Oncology National Cancer Center/National Clinical Research Center for Cancer/Cancer Hospital, Chinese Academy of Medical Sciences and Peking Union Medical College Chaoyang Beijing China; ^3^ Guangxi Medical University Nanning Guangxi China; ^4^ Department of Pathology National Cancer Center/National Clinical Research Center for Cancer/Cancer Hospital, Chinese Academy of Medical Sciences and Peking Union Medical College Chaoyang Beijing China; ^5^ Department of Comprehensive Oncology National Cancer Center/National Clinical Research Center for Cancer/Cancer Hospital, Chinese Academy of Medical Sciences and Peking Union Medical College Chaoyang Beijing China

**Keywords:** angiogenesis inhibitor, chemotherapy, EGFR, exon 20 insertion, immune checkpoint inhibitor, non‐small cell lung cancer

## Abstract

**Background:**

Currently, survival benefit of immunotherapy in advanced non‐small cell lung cancer (NSCLC) with *EGFR* exon 20 insertions (ex20ins) is controversial, though it generally indicates poor response and activity. Compared with standard chemotherapy in combination with bevacizumab, first‐line chemotherapy plus immune checkpoint inhibitor (ICI) in advanced NSCLC with *EGFR* ex20ins remains elusive and lacks real‐world evidence.

**Patients and Methods:**

A retrospective real‐world study was conducted to evaluate clinical outcomes of chemotherapy alone (C), chemotherapy plus ICI (C + I), or chemotherapy plus angiogenesis inhibitors (C + A) as first‐line strategies for advanced NSCLC patients with *EGFR* ex20ins. Investigator‐assessed response and survival outcomes were compared between subgroups. Kyoto Encyclopedia of Genes and Genomes (KEGG) analysis was conducted to reveal concomitant alterations and explore the molecular landscape of ex20ins.

**Results:**

A total of 164 patients were screened, identifying 35 kinds of ex20ins, and 122 cases treated with C, C + I, and C + A were finally included in the first‐line analysis. C + A achieved much better objective response rate (ORR, 38.1% vs. 18.2%) and significant progression‐free survival (PFS) benefit compared with C (median, 7.73 vs.5.93 months, HR = 0.60, 95% CI: 0.40–0.90, *p* = 0.014), and it showed similar ORR (38.1% vs. 40.0%), but higher disease control rate (DCR, 96.8% vs. 80.0%) and numerically longer median PFS (7.73 vs. 6.53 months, HR = 0.83, 95% CI: 0.44–1.56, *p* = 0.30) than C + I. There was no PFS difference between C + I and C, despite of PD‐L1 expression or tumor mutational burden. KEGG analysis revealed concomitant upregulation of *PI3K/AKT* signaling might mediate intrinsic resistance to ICI in ex20ins.

**Conclusion:**

First‐line chemotherapy plus angiogenesis inhibitors might yield more survival benefits than chemotherapy alone for NSCLC with *EGFR* ex20ins, whereas, it suggests that chemotherapy in combination with ICI might not obtain a better survival benefit for this subset of patients. Activation of *PI3K/AKT* signaling might mediate intrinsic immunosuppression in NSCLC with *EGFR* ex20ins.

## Graphical abstract

(1) Chemotherapy plus ICI as first‐line therapy might not yield a better survival benefit than chemotherapy alone or in combination with angiogenesis inhibitors in advanced NSCLC with EGFR ex20ins. (2) Activation of PI3K/AKT signaling might mediate intrinsic resistance to immunotherapy in NSCLC patients with EGFR ex20ins.

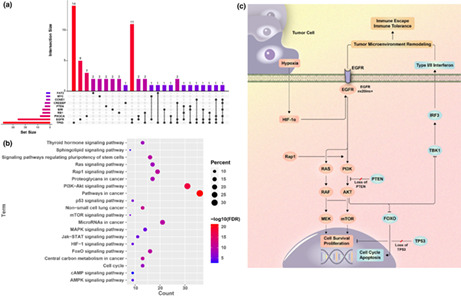

## 1 INTRODUCTION

Tyrosine kinase inhibitor (TKI) for the management of advanced non‐small cell lung cancer (NSCLC) with epidermal growth factor receptor (*EGFR*)‐sensitizing mutations has been established in the first‐line setting, which has become the treatment paradigm for certain *EGFR* aberrations including exon 19 deletions and exon 21 L858R mutations.^1^, ^2^ With the development of genomic sequencing, more uncommon *EGFR* mutations have been identified, in which *EGFR* exon 20 insertions (ex20ins) constitute a heterogeneous subset of *EGFR*‐activating alterations, ranking third with a prevalence of 4%–10% in *EGFR*‐mutant NSCLC.^3^, ^4^, ^5^ Almost 90% of ex20ins variants occur in the loop region following the C‐helix, generally insensitive to clinically approved *EGFR* TKIs owing to their unique conformations and mechanistic differences.^4^, ^6^, ^7^, ^8^, ^9^

In the current international guidelines, platinum‐based chemotherapy alone or in combination with the angiogenesis inhibitor (Ai) bevacizumab is considered as the standard first‐line option for advanced NSCLC with *EGFR* ex20ins.^8^, ^10^, ^11^, ^12^ Recently, specifically designed inhibitors against ex20ins including *EGFR*‐*MET* bispecific monoclonal antibody amivantamab (JNJ‐61186372) and novel inhibitor mobocertinib (TAK‐788) showed high selectivity and potency, and have been approved by the Food and Drug Administration (FDA) of U.S.A. for the treatment of metastatic NSCLC patients with *EGFR* ex20ins failed to prior platinum‐based chemotherapy.^13^, ^14^ In addition, poziotinib, CLN‐081 (TAS6417), and DZD9008 as other promising inhibitors targeting ex20ins are under ongoing clinical trials with favorable activities.^15^, ^16^, ^17^ However, it is currently a dilemma in China for advanced NSCLC patients with *EGFR* ex20ins at the time of disease progression on chemotherapy when no validated targeted agents are available, and what's more, chemotherapy has already hit a plateau.^8^

Immunotherapy with programmed cell death‐1 (PD‐1) or programmed cell death ligand‐1 (PD‐L1) monoclonal antibodies has displayed glorious clinical benefit for wild‐type (WT) advanced NSCLC, but the efficacy of PD‐1/PD‐L1 inhibitor was much limited for patients with oncogenic drivers including *EGFR* mutations or *ALK* fusions.^18^, ^19^ To date, there is no direct evidence on first‐line immunotherapy for advanced NSCLC with *EGFR* ex20ins, even though the post‐analysis of IMpower150 study has indicated clinical benefit of ICI combined with chemotherapy and bevacizumab for this subset of patients.^20^

Although some uncommon *EGFR* mutations were reported to derive clinical benefit from immunotherapy,^21^, ^22^ the correlation between NSCLC with *EGFR* ex20ins and its response to ICI was controversial by several studies, covering a possible trend that ICI might not be a favorable option for ex20ins patients.^8^, ^10^, ^12^, ^22^, ^23^, ^24^, ^25^, ^26^ Yet, there lacks comprehensive study evaluating the exact efficacy of combination therapy with chemotherapy plus Ai or ICI for advanced NSCLC with *EGFR* ex20ins in China. Besides, we acquired little about the heterogeneously molecular landscape of *EGFR* ex20ins in NSCLC. Therefore, we conducted this retrospective real‐world study to compare first‐line clinical outcomes of chemotherapy alone, chemotherapy plus ICI, or chemotherapy plus Ai in advanced NSCLC patients with *EGFR* ex20ins. We also performed bioinformatics analysis to fully investigate the concomitant alterations with ex20ins and explore their molecular landscape.

## 2 PATIENTS AND METHODS

### 2.1 Study design and patients

This real‐world study retrospectively reviewed metastatic NSCLC patients with *EGFR* ex20ins at the National Cancer Center/Cancer Hospital, Chinese Academy of Medical Sciences between December 2015 and September 2021. The inclusion criteria were as follows: (1) Stage IV disease at initial diagnosis; (2) ≥18 years of age; (3) histologically or cytologically confirmed NSCLC; (4) *EGFR* ex20ins mutations confirmed at initial diagnosis by next‐generation sequencing (NGS) or polymerase chain reaction (PCR) with tumor tissues or liquid biopsy samples; (5) documented with available data of first‐line therapies in medical records. PD‐L1 expression was detected by Dako 22C3 pharmDx test kit. The PD‐L1 tumor proportion score (TPS) was calculated as the percentage of ≥100 viable tumor cells with complete or partial membrane staining. Tumor mutational burden (TMB), defined as the number of somatic, coding base substitutions, and short insertions and deletions per megabase of genome examined, was assessed by formalin‐fixed, paraffin‐embedded tissue samples or blood using NGS method covering various gene panels. The NGS testing in this study was performed in institutional laboratories or qualified third‐party genetic testing companies as documented in medical records, which had acquired the national quality system certification in China, and all of the NGS testings were carried out based on the Illumina sequencing system. All clinical data were extracted from electronic records. This retrospective study was approved by the Ethics Committee of National Cancer Center/Cancer Hospital, Chinese Academy of Medical Sciences, and written informed consent was waived.

### 2.2 Treatment and assessment

Patients who met the above inclusion criteria were collected for molecular analysis, and those who were treated with different therapy patterns of chemotherapy alone (C), chemotherapy plus Ai (C + A), or chemotherapy plus ICI (C + I) were included for first‐line efficacy analysis. Patients receiving the above treatment options were at a standard dose according to clinical guidelines in practice. Baseline images of measurable target lesions were obtained with computed tomography of the chest and abdomen, and the magnetic resonance imaging of brain. Guideline from the Response Evaluation Criteria in Solid Tumors (RECIST) version 1.1 was performed to identify a response of complete response (CR), partial response (PR), stable disease (SD), or progressive disease (PD). Progression‐free survival (PFS) was defined as the time from initiation of first‐line therapy to date of documented disease progression or death from any cause. The objective response rate (ORR) was calculated as the percentage of confirmed CR and PR. Disease control rate (DCR) was defined as the percentage of CR, PR, and SD. Overall Survival (OS) was the time from initiation of first‐line therapy to death. All these endpoints were assessed by investigators responsible for this study.

### 2.3 Statistical analysis

Statistical analyses were conducted using SPSS version 23.0 (SPSS Inc.). Continuous variables were summarized using medians and ranges, and categorical variables were described using frequency and percentage. Comparison among the subgroups was performed using analysis of variance or Chi‐Square test accordingly. PFS and OS were analyzed using the Kaplan–Meier method. PFS between different subgroups were compared using the log‐rank test (two‐sided), and the corresponding hazard ratio (HR) and 95% confidence interval (CI) were estimated using the Cox proportional regression model. *p* < 0.05 was considered statistically significant. *p* values for these analyses are nominal, and all are two‐sided. The Kyoto Encyclopedia of Genes and Genomes (KEGG) pathway enrichment analysis was performed in R version 4.0.5 with DAVID version 6.8 to reveal differentially expressed concomitant mutations with *EGFR* ex20ins.

## 3 RESULTS

### 3.1 Patient characteristics

As the flow chart showed (Figure S1), a total of 164 NSCLC patients who met the above inclusion criteria were screened. Excluded for 38 patients initiated with *EGFR* TKIs, one patient with chemotherapy plus TKI, and 3 patients with ICI single agent or plus Ai, finally, 122 cases receiving chemo‐based therapies were included for first‐line analysis. Among them, 59.8% (*n* = 73) were females, 69.7% (*n* = 85) were never‐smokers, 20.5% (*n* = 25), and 7.4% (*n* = 9) presented with baseline central nervous system (CNS) and liver metastases, respectively. Almost all of them (*n* = 116, 95.1%) received genomic testing with tumor tissues, and 89.3% (*n* = 109) were treated with pemetrexed/platinum chemotherapy. In subgroup C, most of the patients (86.4%) received regimens of pemetrexed /platinum (including cisplatin, carboplatin, and nedaplatin), and five patients were treated with paclitaxel/platinum, with one of gemcitabine/cisplatin. In subgroup C + A, 58 patients (92.1%) received regimens of pemetrexed /platinum plus Ai (52 cases with bevacizumab, three cases with *VEGFR2*‐targeted TKI apatinib, and two with recombinant human endostatin). Three patients received paclitaxel/platinum plus bevacizumab, and two were treated with docetaxel/platinum plus bevacizumab. In subgroup C + I, 13 patients (86.7%) were treated with pemetrexed/platinum plus PD‐1 or PD‐L1 inhibitors, and two patients received nab‐paclitaxel/carboplatin plus pembrolizumab. Among 15 patients receiving C + I, except for one case with pemetrexed/carboplatin plus PD‐L1 inhibitor atezolizumab, the others all received platinum‐based chemotherapy plus PD‐1 inhibitors including pembrolizumab (*n* = 9), sintilimab (*n* = 3), camrelizumab (*n* = 1), and toripalimab (*n* = 1). Baseline clinicopathological characteristics of *EGFR* ex20ins patients receiving chemo‐based therapies in first‐line setting was demonstrated in Table 1.

**TABLE 1:** Clinicopathological characteristics of *EGFR* exon 20 insertion patients in the first‐line setting

Characteristics	C (*n* = 44)	C + A (*n* = 63)	C + I (*n* = 15)	Overall (*N* = 122)	*p*‐value
Age (years)	54.8 ± 8.41	52.7 ± 10.60	51.4 ± 9.36	53.3 ± 9.73	0.39
Gender					
Male	17 (38.6%)	26 (41.3%)	6 (40.0%)	49 (40.2%)	0.97
Female	27 (61.4%)	37 (58.7%)	9 (60.0%)	73 (59.8%)	
Pathology					0.12
Adenocarcinoma	41 (93.2%)	62 (98.4%)	13 (86.6%)	116 (95.0%)	
Squamous carcinoma	2 (4.5%)	0 (0%)	1 (6.7%)	3 (2.5%)	
Adenosquamous carcinoma	1 (2.3%)	1 (1.6%)	1 (6.7%)	3 (2.5%)	
Smoking history					0.76
Never	30 (68.2%)	43 (68.3%)	12 (80.0%)	85 (69.7%)	
Current/former	14 (31.8%)	20 (31.7%)	3 (20.0%)	37 (30.3%)	
Brain metastases					0.74
Absence	36 (81.8%)	50 (79.4%)	11 (73.3%)	97 (79.5%)	
Presence	8 (18.2%)	13 (20.6%)	4 (26.7%)	25 (20.5%)	
Liver metastases					0.79
Absence	42 (95.5%)	57 (90.5%)	14 (93.3%)	113 (92.6%)	
Presence	2 (4.5%)	6 (9.5%)	1 (6.7%)	9 (7.4%)	
NGS specimen					0.72
Tumor tissue	41 (93.2%)	60 (95.2%)	15 (100%)	116 (95.1%)	
Plasma	3 (6.8%)	3 (4.8%)	0 (0.0%)	6 (4.9%)	
TP53 mutation					0.06
None	8 (18.2%)	10 (15.9%)	2 (13.3%)	20 (16.4%)	
Yes	5 (11.4%)	19 (30.2%)	7 (46.7%)	31 (25.4%)	
NA	31 (70.4%)	34 (54.0%)	6 (40.0%)	71 (58.2%)	
PD‐L1 expression^†^					0.02
Negative	4 (9.1)	9 (14.3%)	2 (13.3%)	15 (12.3%)	
1 ≤ TPS < 50%	4 (9.1%)	4 (6.3%)	6 (40.0%)	14 (11.5%)	
TPS≥50%	2 (4.5%)	2 (3.2%)	1 (6.7%)	5 (4.1%)	
NA	34 (77.3%)	48 (76.2%)	6 (40.0%)	88 (72.1%)	
TMB value (Mb/Muts)					0.86
<10	4 (9.1%)	9 (14.3%)	1 (6.7%)	14 (11.5%)	
≥10	2 (4.5%)	2 (3.2%)	0 (0.0%)	4 (3.3%)	
NA	38 (86.4%)	52 (82.5%)	14 (93.3%)	104 (85.2%)	
Chemotherapy regimens					0.16
Platinum/pemetrexed	38 (86.4%)	57 (90.5%)	10 (66.7%)	105 (86.1%)	
Other regimens	6 (13.6%)	6 (9.5%)	5 (33.3%)	17 (13.9%)	

Abbreviations: C, chemotherapy alone; C + A, chemotherapy plus angiogenesis inhibitors; C + I, chemotherapy plus immune checkpoint inhibitors; NA, not available; TMB, tumor mutational burden; TPS, tumor proportion score.

^†^ There were no differences among the groups except PD‐L1 expression (*p* = 0.02).

### 3.2 Molecular subtypes of exon 20 insertions

**FIGURE 1:**
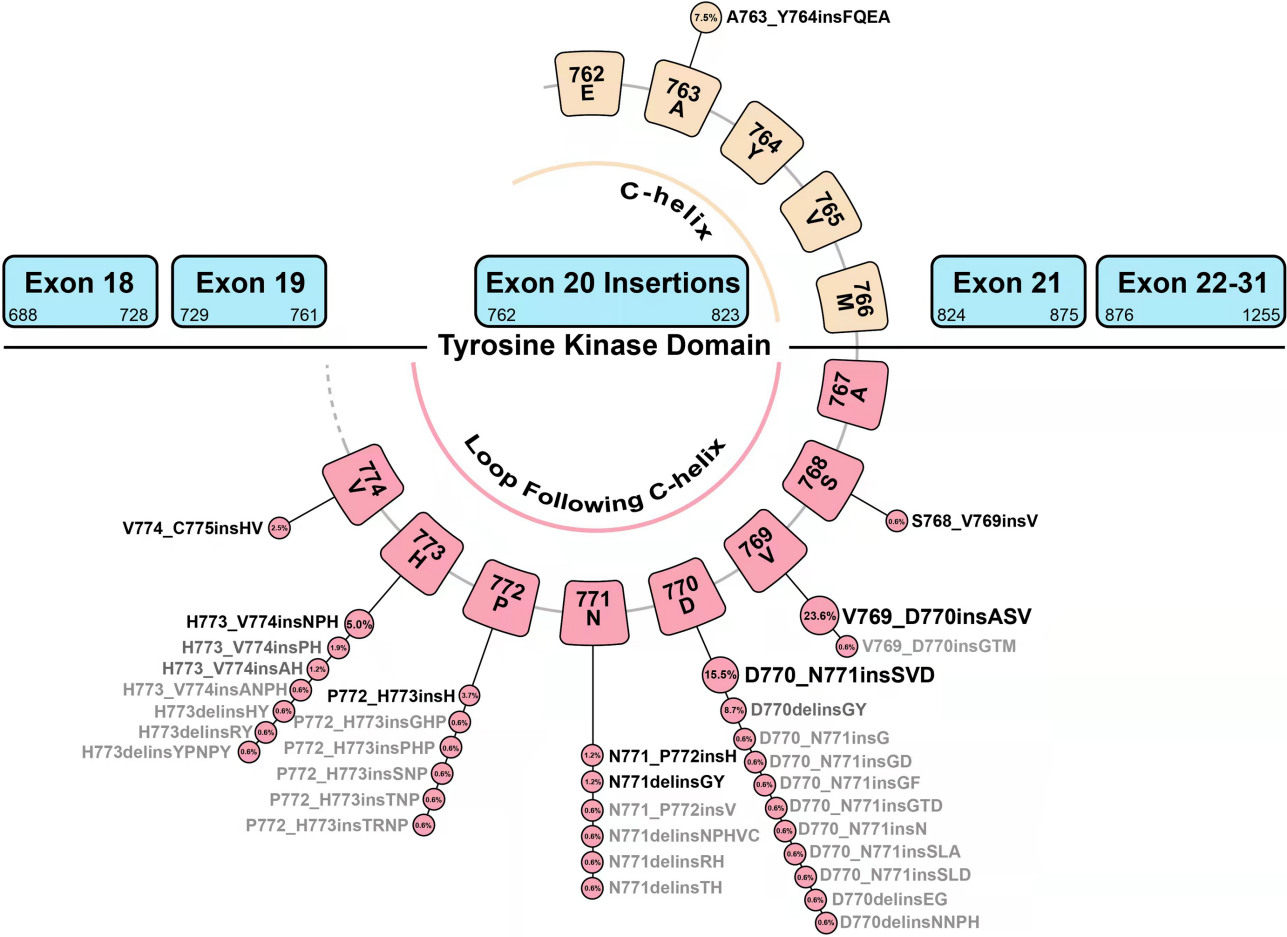
Molecular subtypes of NSCLC patients with distinct EGFR exon 20 insertions

Among 161 patients who had received NGS testing for ex20ins detection at initial diagnosis, it identified 35 kinds of distinct ex20ins subtypes, in which V769_D770insASV (A767_V769dup) was the most common variant (*n* = 38, 23.6%), followed by D770_N771insSVD (S768_D770dup, *n* = 25, 15.5%), D770delinsGY (*n* = 14, 8.7%), A763_Y764insFQEA (*n* = 12, 7.5%), H773_V774insNPH (N771_H773dup, *n* = 8, 5.0%), P772_H773insH (H773dup, n = 6, 3.7%), V774_C775insHV (H773_V774dup, *n* = 4, 2.5%), and other subtypes (*n* = 33, 20.5%). In addition, 3 patients (1.8%) were identified to be ex20ins positive by PCR assay, with uncertain insertion subtypes (Figure 1).

### 3.3 Efficacy

**FIGURE 2:**
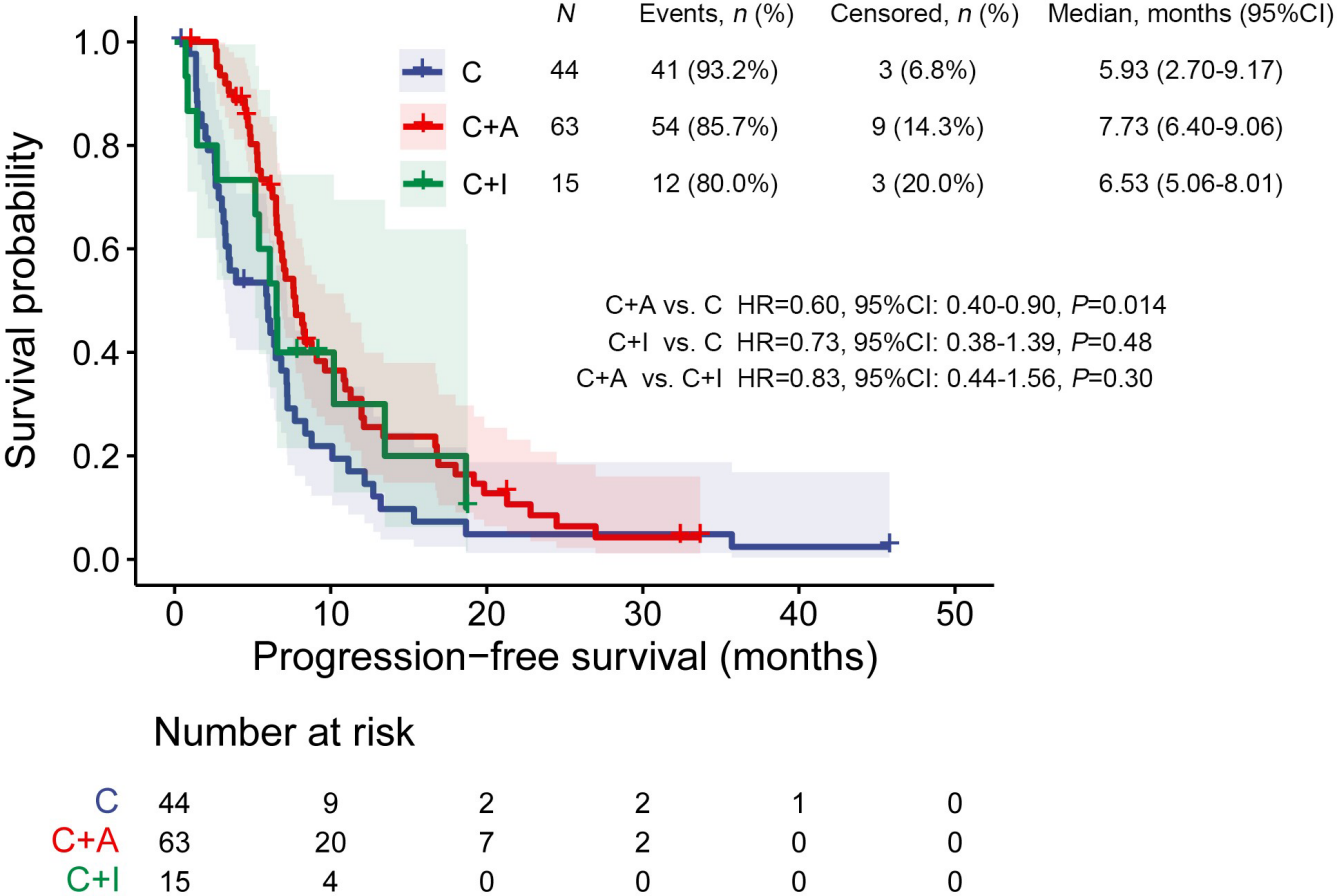
Kaplan–Meier curves of PFS in patients harboring EGFR ex20ins with chemotherapy alone (C) versus chemotherapy plus angiogenesis inhibitors (C + A) versus chemotherapy plus ICI (C + I) as first‐line therapies

The cutoff time of this study was September 30, 2021, with a median follow‐up of 32.40 months. One‐hundred and twenty‐two patients were available for first‐line efficacy analysis, and 107 had PFS events. The ORR, DCR, median PFS, and median OS of C were 18.2% (8/44), 84.1% (37/44), 5.93 months (95% CI: 2.70–9.17), and 32.03 months (95% CI: 17.55–46.52), respectively. Sixty‐three patients (51.6%) were treated with C + A, and the ORR, DCR, median PFS, and median OS were 38.1% (24/63), 96.8% (61/63), 7.73 months (95% CI: 6.40–9.06), and 30.57 months (95% CI: 19.90–41.23), respectively. Fifteen patients (12.3%) administered C + I, and the ORR, DCR, and median PFS were 40.0% (6/15), 80.0% (12/15), and 6.53 months (95% CI: 5.06–8.01), respectively. At the cutoff time, the median OS of C + I was immature because of this treatment pattern was used in more recent times with only three events of deaths, but not for better efficacy. The PFS by the above three treatment patterns was listed in Figure 2.

It was observed significant PFS difference between C + A and C (median, 7.73 vs. 5.93 months, HR = 0.60, 95% CI: 0.40–0.90, *p* = 0.014). No difference in PFS was observed between C + I and C (median, 6.53 vs.5.93 months, HR = 0.73, 95% CI: 0.38–1.39, *p* = 0.48), or C + A and C + I (median, 7.73 vs.6.53 months, HR = 0.83, 95% CI: 0.44–1.56, *p* = 0.30). Besides, we also analyzed the impact of metastatic organs on efficacy. However, the patients who were diagnosed with baseline CNS metastases did not identify an inferior PFS outcome by first‐line chemo‐based therapies (median, 7.20 vs. 6.73 months, HR = 0.90, 95% CI: 0.57–1.44, *p* = 0.66). And in the patients with baseline liver metastases, we observed similar PFS trend (median, 6.50 vs. 6.90 months, HR = 1.50, 95% CI: 0.72–3.09, *p* = 0.27).

### 3.4 PD‐L1 and TMB as biomarkers for efficacy evaluation of ICI

Among 15 patients receiving C + I as first‐line treatment and available for efficacy evaluation, nine cases had molecular data of PD‐L1 expression. For two patients with TPS negative, the PFS was 6.53 and 7.83 months. Among another six patients who carried 1 ≤ TPS < 50%, the ORR was 33.3% (2/6), with a median PFS of 6.57 months (95% CI: 0.53–12.61). There was only one patient with TPS of 60%, and he achieved PR with a PFS of 5.40 months. For five patients who detected TMB values before first‐line therapy, one with TMB of 2.10 Muts/Mb showed SD to PD‐1 inhibitor with an ongoing PFS of 6.53 months. The other four patients all presented TMB < 10, and the PFS were 10.27, 4.10, 6.03, and 2.43 months, respectively. Their responses to C + I were SD, SD, SD, and PR, respectively.

### 3.5 Genomic analysis for concomitant alterations with exon 20 insertions

**FIGURE 3:**
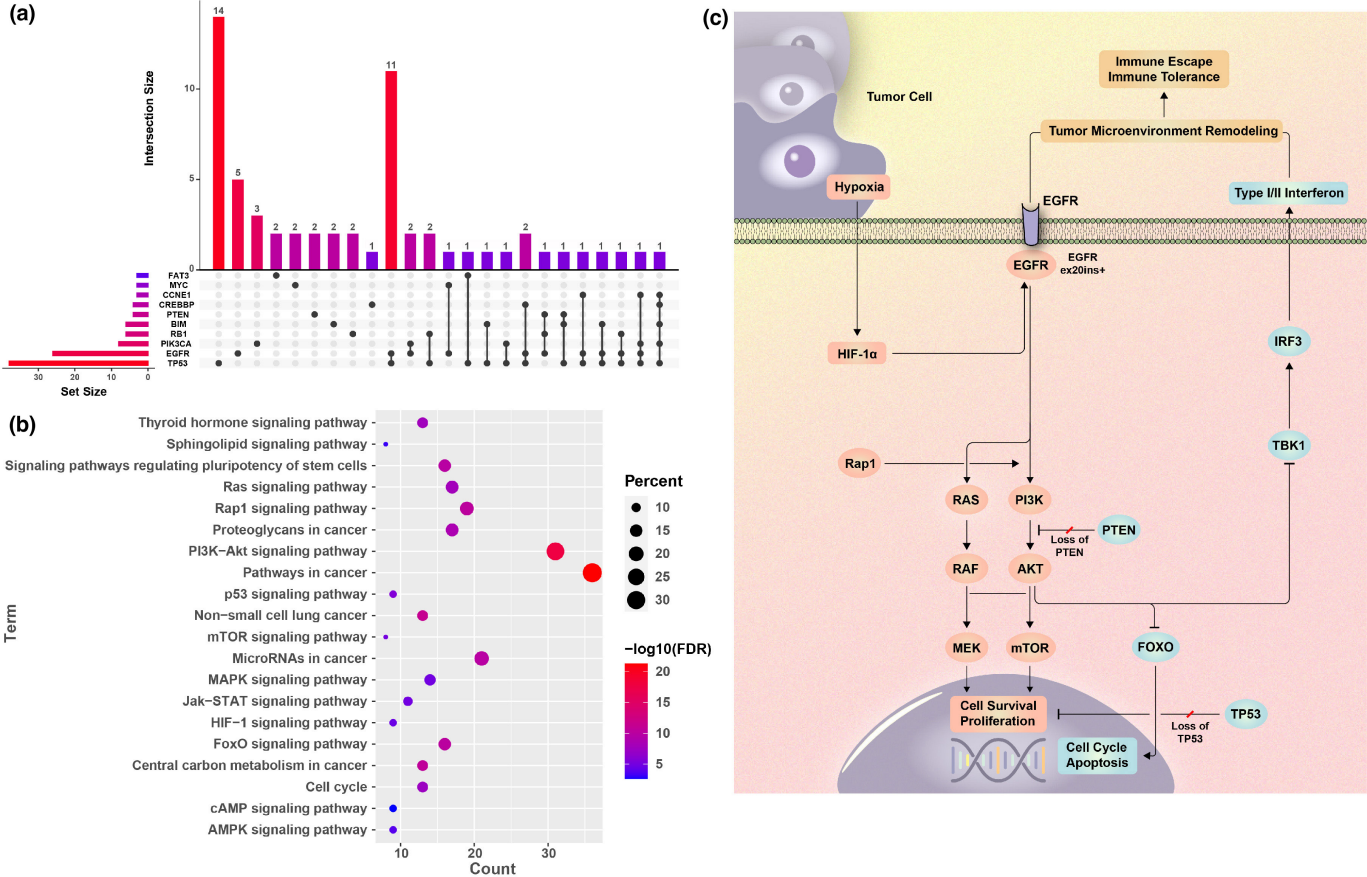
The top 10 concomitant genes together with EGFR ex20ins in NSCLC patients detected at primary diagnosis in the UpSet plot (A). KEGG analysis of concomitant signaling pathways detected in patients with EGFR ex20ins (B). Possible mechanism for immunosuppression in NSCLC with EGFR ex20ins mediated by activation of PI3K/AKT signaling (C)

The UpSet plot was constructed for the interactive sets between the top 10 concomitant genes together with ex20ins among 73 patients (45.3%) at initial diagnosis (Figure 3A). The most frequent co‐mutation identified in this study was *TP53* aberration (*n* = 38, 52.1%), followed by *EGFR* amplification (*n* = 26, 35.6%), mutations of *PIK3CA* (*n* = 8, 11.0%) and *RB1* (*n* = 6, 8.2%), *BIM* deletion polymorphism (*n* = 6, 8.2%), alterations of *PTEN* (*n* = 4, 5.5%) and *CREBBP* (*n* = 4, 5.5%).

In addition, a total of 112 genes were detected in ex20ins patients and involvement of these genes were overlaid on the 20 significant KEGG pathways (Figure 3B). The genes were significantly enriched in cancer‐related pathways, *PI3K/AKT*, microRNAs, *Rap1* signaling pathway, proteoglycans, cell cycle genes, and so on.

## 4 DISCUSSION

This study provided comprehensive real‐world evidence regarding clinical efficacy of chemo‐based combination therapies for advanced NSCLC with *EGFR* ex20ins alterations in the first‐line setting in China. We examined heterogeneous ex20ins subtypes and revealed full‐scale concomitant alterations with *EGFR* ex20ins.

Notably, this is the first Chinese study to directly compare the clinical outcomes of ICI or Ai in combination with chemotherapy as first‐line strategies for advanced NSCLC with *EGFR* ex20ins. We observed a much better ORR (38.1% vs. 18.2%) and significant PFS benefit (median, 7.73 vs. 5.93 months, HR = 0.60, *p* = 0.014) of C + A compared with chemotherapy alone, which was much similar to that reported in our previous nationwide study, indicating a median PFS of 7.5 and 5.6 months respectively for NSCLC patients with *EGFR* ex20ins.^8^ In addition, C + A showed similar ORR (38.1% vs. 40.0%), but achieving a higher disease control rate (DCR, 96.8% vs. 80.0%) and numerically longer PFS (median, 7.73 vs. 6.53 months, HR = 0.83, 95% CI: 0.44–1.56, *p* = 0.30) compared with C + I. There was no PFS difference on C + I compared with C or C + A in first‐line setting. Similarly, series of studies reported worldwide also issued that ICI plus chemotherapy failed to improve survival benefits for ex20ins patients compared with conventional chemotherapy,^10^, ^12^, ^23^, ^25^, ^28^ with an overall ORR of 6.7%–33.3%, and median PFS of 2.0–7.0 months (Table 2). Especially, ICI monotherapy was reported with much limited activity for ex20ins, with an overall ORR of 3.1%–50%, and PFS of 1.9–4.8 months.^10^, ^12^, ^22^, ^24^, ^26^, ^27^, ^29^, ^30^, ^31^, ^32^

**TABLE 2:** Reported studies of ICI for advanced NSCLC with *EGFR* exon 20 insertion

Author	Year	Sample size*	Therapeutics	ORR (%)	DCR (%)	Median PFS (months)	Median OS (months)
Negrao et al.^26^	2018	36 (36)	ICIs	25	50	2.9	NR
Takeda et al.^29^	2018	5 (12)	PD‐1 inhibitor	20	40	NA	NA
Hastings et al. ^22^	2019	28 (28)	PD‐1/PD‐L1 ± CTLA‐4 inhibitors	10.7	32.1	1.9	5.5
Dersarkissian et al.^31^	2019	29 (199)	ICIs	NA	NA	NA	NA (range, 6.1–8.0)
Yang et al.^8^	2020	10 (165)	ICIs ± PbC or Ai	50	80	NA (range, 1.5–8.7)	NA
Patil et al.^28^	2020	3 (38)	ICIs ± PbC	33.3	NA	NA	NA
Tomaras et al.^32^	2020	30 (NA)	ICIs	10.0	NA	3.2	7.5
Choudhury et al.^10^	2021	15 (59)	PD‐1 inhibitor	NA	NA	2.8	NA
		12 (59)	PD‐1 inhibitor + PbC	NA	NA	7.0	NA
Ou et al.^12^	2021	11 (237)	ICIs	9.1	NA	3.1	11.1
		16 (237)	ICIs + PbC	18.8	NA	4.5	11.3
		32 (237)	ICIs	3.1	NA	2.3	8.1
Metro et al.^23^	2021	15 (30)	ICIs ± PbC	6.7	13.4	2.0	5.3
Chen et al.^24^	2021	9 (35)	ICIs	22.2	NA	4.0	23.3
Geng et al.^25^	2021	12 (283)	PD‐1 inhibitor ± PbC or Ai	16.7	83.3	NA (range, 1.5–5.0)	NA
Lau et al.^27^	2021	6 (6)	PD‐1/PD‐L1 inhibitors	50	67	4.8	NA
Morita et al.^30^	2021	8 (23)	PD‐1 inhibitor	25	50	3.1	NA

Abbreviations: Ai, angiogenesis inhibitors; CTLA‐4, cytotoxic T lymphocyte‐associated antigen‐4; DCR, disease control rate; NA, not available; NR, not reached; ICIs, immune checkpoint inhibitors; ORR, overall response rate; OS, overall survival; PbC, platinum‐based chemotherapy; PD‐1, programmed cell death protein 1; PD‐L1, programmed cell death ligand 1; PFS, progression‐free survival.

* Expressed as patient number with *EGFR* ex20ins treated with immunotherapy (total patient number with *EGFR* ex20ins).

Our study identified and reported 35 different kinds of heterogeneous ex20ins subtypes, in which V769_D770insASV and D770_N771insSVD were the most common insertion variants, overall representing 40% of all ex20ins subtypes. This finding was in accordance with two previous large sample Chinese studies,^8^, ^25^ and also was similar to that discovered in the American databases.^33^, ^34^ Similarly, we also found *TP53* occurred as the most frequent concomitant alteration together with *EGFR* ex20ins, with a prevalence of 52.1% in this study, which was much alike with those of 35%–56% reported by other studies.^10^, ^11^, ^23^, ^24^, ^25^, ^33^, ^35^ As clarified by current reports, *EGFR* ex20ins was associated with much lower levels of TMB (2.8–3.6/Muts/Mb) than WT *EGFR*, as well as with a low TPS of PD‐L1 expression, which suggested lack of comparable benefit of ICI in *EGFR* ex20ins as observed in NSCLC harboring common *EGFR* mutations.^10^, ^21^, ^22^, ^33^ In our study, only 2 patients carrying 1 ≤ TPS˂50% showed response to C + I (ORR, 2/6), with a median PFS of 6.57 months. Five patients available for TMB assessment all harbored low TMB values less than 10 Muts/Mb, and showed ORR of 1/5 to C + I, with PFS between 2.43 and 10.27 months. Outcomes of C + I observed in our study were well correlated with previous observations reported worldwide, indicating a limited activity of ICI for NSCLC patients with *EGFR* ex20ins.^8^, ^10^, ^12^, ^22^, ^23^, ^24^, ^25^, ^26^, ^27^, ^28^, ^29^, ^30^, ^31^, ^32^

To date, it lacks in vitro and molecular exploration for the possible mechanism of unfavorable activity of ICI for *EGFR* ex20ins. In this study, we observed concomitant genes with ex20ins significantly enriched in *PI3K/AKT*, microRNAs in cancer, and *Rap1* signaling pathways by KEGG analysis, revealing the upregulation of *PI3K/AKT* signaling was much more common and important in NSCLC with *EGFR* ex20ins, which had been reported in *HER2*‐positive breast cancer.^36^ Recent studies have confirmed that microRNAs are involved in tumor proliferation and chemotherapy resistance in NSCLC.^37^, ^38^ The dysregulation of microRNAs is closely related to the remodeling of tumors and the immune microenvironment.^39^ *EGFR* has a regulatory role in microRNAs maturation through phosphorylation of *AGO2* with *HIF‐1α* gene expression.^40^, ^41^ At the meantime, *TP53* can modulate cell survival and proliferation by interacting with microRNAs.^38^ *Rap1* plays an important role in the cell behaviors by regulating the function of integrins and other adhesion molecules in different cell types, with promoting *AKT* phosphorylation.^42^

Based on the annotation of the KEGG website (http://www.kegg.jp) and pathway enrichment analysis in this study, we supposed that together with the activation of *Rap1* and the loss of *PTEN* could enhance *PI3K/AKT* signaling and ultimately suppress the activation of *FOXO* proteins, thus mediating drug resistance, including poor response to ICI in *EGFR* ex20ins. In terms of possible mechanisms explaining immunosuppressive microenvironment in NSCLC with *EGFR* ex20ins, we explained that ex20ins could activate the downstream *MAPK* and *PI3K/AKT* signaling pathways, resulting in *AKT1* recruitment and impairing *TANK*‐binding kinase 1 (*TBK1*) phosphorylation, and suppress interferon regulatory factor 3 (*IRF3*), thus disrupting the stimulator of interferon gene (*STING*) signaling and suppressed type I and II interferon response and antitumor immune responses (Figure 3C), which had been currently reported by in vitro researches.^43^, ^44^

This study has several limitations. First, it inevitably exists selective bias due to the retrospective nature. In addition, although this study provides comprehensive subtypes of *EGFR* ex20ins with detailed concomitant alterations in several signaling pathways, less than half of the patients received large panels of NGS testing, with not adequate data for molecular analysis. Further, although we demonstrated ICI in combination with chemotherapy might not improve survival outcomes over chemotherapy alone or plus angiogenesis inhibitors in NSCLC with *EGFR* ex20ins, corresponding results should be interpreted with caution due to the small sample size receiving immunotherapy in this study. As well, the lack of in vitro evidence on tumor immunohistochemistry or flow cytometry data of *PI3K/AKT* pathway and other immunosuppressive genes among *EGFR* ex20ins patients were considered another limitation.

In conclusion, chemotherapy plus ICI as first‐line therapy might not yield a better survival benefit than chemotherapy alone or in combination with angiogenesis inhibitors in advanced NSCLC with *EGFR* ex20ins. *PI3K/AKT* signaling might mediate intrinsic resistance to immunotherapy in this subset of patients. New therapeutics targeting the immunosuppressive microenvironment in NSCLC with *EGFR* ex20ins need to be explored.

## AUTHOR CONTRIBUTIONS

Guangjian Yang, Yaning Yang, and Runze Liu: conceptualization, methodology, software, formal analysis, project administration, data curation, writing ‐ original draft, writing ‐ review & editing. Weihua Li, Haiyan Xu, Xuezhi Hao, and Junling Li: validation, resources, visualization. Shuyang Zhang, Fei Xu, and Siyu Lei: investigation. Yan Wang: conceptualization, resources, supervision, writing ‐ review & editing.

## CONFLICT OF INTEREST

The authors declare no conflict of interest.

## ETHICS STATEMENT

As an observational study, this research was exempted from obtaining patients' informed consent without therapeutic intervention, and this research was approved by the Research Ethics Board of Cancer Hospital and conducted in accordance with the Declaration of Helsinki as well.

## Supporting information


Figure S1

## Data Availability

Data supporting the results presented in this study are available from the corresponding author upon reasonable request.
