# Supplementary figures and images for: First‐line immunotherapy or angiogenesis inhibitor combined with chemotherapy for advanced non‐small cell lung cancer with EGFR exon 20 insertions: Real‐world evidence from China
Source: Cancer Med. 2022 May 24;12(1):335–44. doi: 10.1002/cam4.4852 (PMC9844624; doi:10.1002/cam4.4852)

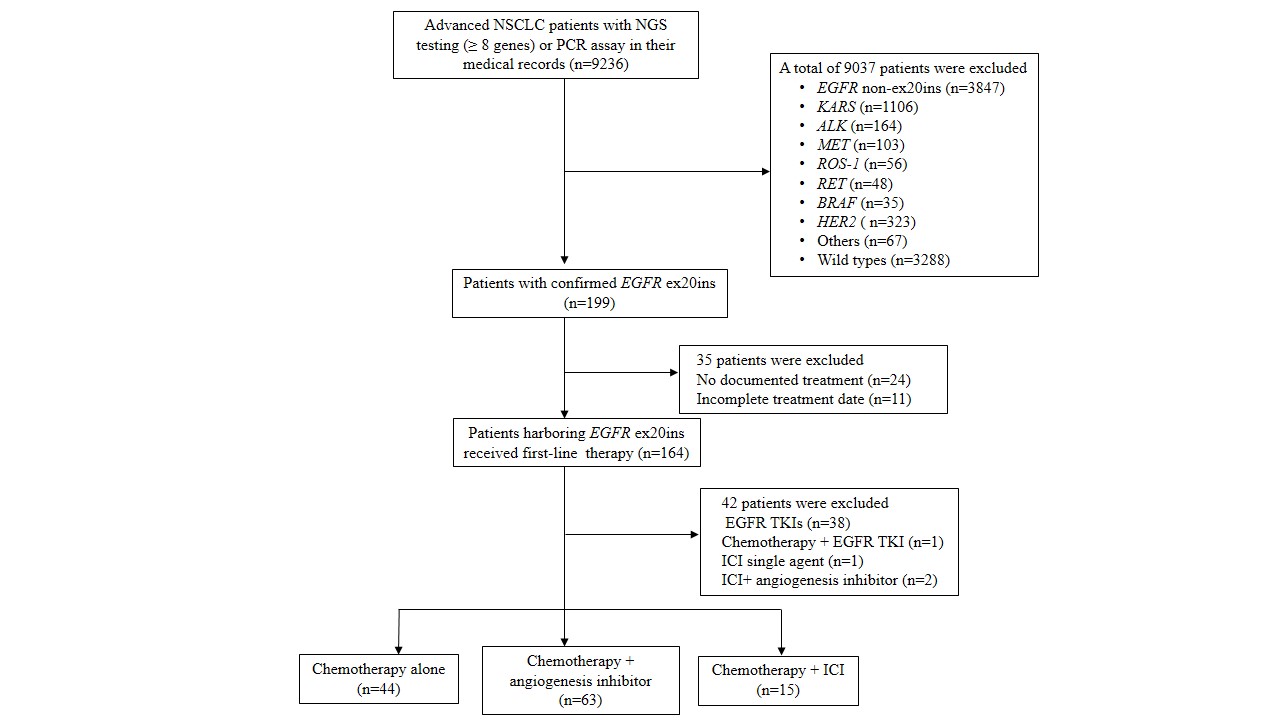

Supplement: Supplementary file 1 — Figure S1 [file CAM4-12-335-s001.jpg]
